# Gene Therapy in Hemophilia: Recent Advances

**DOI:** 10.3390/ijms22147647

**Published:** 2021-07-17

**Authors:** E. Carlos Rodríguez-Merchán, Juan Andres De Pablo-Moreno, Antonio Liras

**Affiliations:** 1 Osteoarticular Surgery Research, Hospital La Paz Institute for Health Research–IdiPAZ (La Paz University Hospital—Autonomous University of Madrid), 28046 Madrid, Spain; ecrmerchan@hotmail.com; 2 Department of Genetic, Physiology and Microbiology, Biology School, Complutense University of Madrid, 28040 Madrid, Spain; jdepablo@ucm.es

**Keywords:** hemophilia, advanced therapies, gene therapy, FVIII transgene, FIX transgene, adeno-associated virus, lentiviral vectors

## Abstract

Hemophilia is a monogenic mutational disease affecting coagulation factor VIII or factor IX genes. The palliative treatment of choice is based on the use of safe and effective recombinant clotting factors. Advanced therapies will be curative, ensuring stable and durable concentrations of the defective circulating factor. Results have so far been encouraging in terms of levels and times of expression using mainly adeno-associated vectors. However, these therapies are associated with immunogenicity and hepatotoxicity. Optimizing the vector serotypes and the transgene (variants) will boost clotting efficacy, thus increasing the viability of these protocols. It is essential that both physicians and patients be informed about the potential benefits and risks of the new therapies, and a register of gene therapy patients be kept with information of the efficacy and long-term adverse events associated with the treatments administered. In the context of hemophilia, gene therapy may result in (particularly indirect) cost savings and in a more equitable allocation of treatments. In the case of hemophilia A, further research is needed into how to effectively package the large factor VIII gene into the vector; and in the case of hemophilia B, the priority should be to optimize both the vector serotype, reducing its immunogenicity and hepatotoxicity, and the transgene, boosting its clotting efficacy so as to minimize the amount of vector administered and decrease the incidence of adverse events without compromising the efficacy of the protein expressed.

## 1. Introduction

Hemophilia A (HA) and hemophilia B (HB) are X-linked hereditary hemorrhagic disorders arising from mutations in the genes encoding coagulation factors VIII (FVIII) in HA and IX (FIX) in HB [1]. HA, with a prevalence of one in every 5000 live births, is more common than HB, which affects one in every 30,000 live births.

The severity of HA and HB is contingent on the functional levels of the corresponding circulating factors, with levels of FVIII or FIX below 1% considered diagnostic of severe hemophilia, levels between 1 and 5% diagnostic of moderate hemophilia and those between 5% and 40% of mild hemophilia.

Patients with severe hemophilia tend to experience bleeds into their joints, muscles or soft tissues following trauma or even without any apparent cause. They may also suffer life-threatening hemorrhagic episodes such as intercranial bleeding. Persons with mild or moderate factor deficiencies may experience spontaneous bleeding, with excessive bleeds occurring only after trauma or an invasive medical procedure.

Residual factor activity is usually well correlated with patients’ clinical characteristics. However, individuals with the same coagulation factor levels may exhibit varying bleeding phenotypes. Although HA and HB are considered clinically indistinguishable, several recent studies have questioned this idea, suggesting that patients with HB could be less prone to severe bleeds than those with HA with the same plasma levels of residual factor (identical disease phenotypes) [2].

Treatment of hemophilia [3] has evolved very rapidly in the last few decades since the advent of plasma and cryoprecipitates; human plasma-derived clotting factor concentrates, and more recently recombinant factors, characterized by a high degree of purity and a longer half-life. These innovations have allowed the development of extremely safe against emerging pathogens and highly efficient treatments based on the replacement of the deficient clotting factor. Treatment protocols consist of intravenous administration of the deficient clotting factor either on-demand upon occurrence of bleeding episodes or prophylactically two or three times a week. Although they are generally highly effective, these replacement treatments may occasionally fail due to the presence of the so-called inhibitors present in up to 30% of hemophiliacs. Inhibitors are neutralizing antibodies that target exogenously administered coagulation factors.

Next-generation recombinant products offer a longer half-life, which allows for less frequent dosing and therefore higher adherence to treatment and a better quality of life. Recombinant FVIII/FIX concentrates are obtained by fusion to polyethylene glycol, IgG1-Fc or albumin [4].

New treatment strategies have recently been developed as alternatives to replacement protocols. One of these is emicizumab (Hemlibra^®^), a subcutaneously administered bispecific monoclonal antibody recently approved for treatment of HA with or without inhibitors against FVIII. This antibody binds to activated factor X (FXa) and to activated FIX (FIXa) simulating the function of FVIII. Given emicizumab’s unique structure, its effect is not likely to be neutralized by inhibitors against FVIII. HAVEN 1, a non-interventional phase 3 trial involving patients with severe HA and inhibitors, demonstrated that patients on emicizumab had 87% less bleeding episodes than those without prophylaxis, and 79% less bleeding episodes than those on previous prophylaxis [5].

On the other hand, recent studies have shown that the natural anticoagulation pathway (antithrombin and tissue factor pathway inhibitors, protein S and protein C) is capable of restoring hemostasis in patients with a bleeding disorder [6]. Fitusiran is an RNA interference (RNAi) therapy that targets antithrombin (AT) in the liver and interferes with the translation of its messenger RNA, preventing AT synthesis and promoting hemostasis. Preclinical and clinical studies have found that AT suppression leads to a dose-dependent reduction in AT levels which results in a decreased severity of the bleeding phenotypes in patients with hemophilia [7,8]. Several phase 1 and phase 2 studies have tested the effectiveness of Fitusiran in patients with hemophilia with or without inhibitors; phase 3 trials are now underway with the same goal in mind. A phase 1 clinical trial (NCT02035605) demonstrated a 61–89% dose-dependent decrease in AT, which was correlated with increased thrombin generation in patients with HA and patients with HB without inhibitors.

Research is at present also focusing on the tissue factor pathway inhibitor (TFPI), the main inhibitor of the onset of the coagulation cascade, which has been shown to regulate the severity of a wide range of hemorrhagic and coagulation disorders. Multiple animal studies have shown that TFPI inhibition can reduce bleeding in the context of hemophilia [9]. In this respect, several strategies have been described with a view to inhibiting TFPI by means of aptamers, fucoidan, monoclonal antibodies and peptide agents [10]. Concizumab, a humanized monoclonal antibody targeted against TFPI, is the most highly developed of these strategies. Two phase 2/3 clinical trials are currently underway in patients with HA and HB with or without inhibitors. Specifically, TFPI inhibits activated FX (FXa) while activated protein C (APCa) degrades activated factor V (FVa) and activated FVIII (FVIIIa). APC is a serine protease that can bind to the endothelial protein C receptor, which approximates it to thrombin for its activation. This means that it acts by decreasing the amplification of FXa generation and intrinsic thrombin generation by FVa and FVIIIa proteolysis. APC is regulated by serine protease inhibitors (serpins), the protein C inhibitor (PCI), protein S and α1-antitrypsin. Available non-clinical data support the use of a serpin (serpinPC), a monoclonal antibody against APC, and protein S-targeted siRNA. SerpinPC is currently in the early stages of clinical investigation.

## 2. Advanced Therapies in Hemophilia

**Figure 1:**
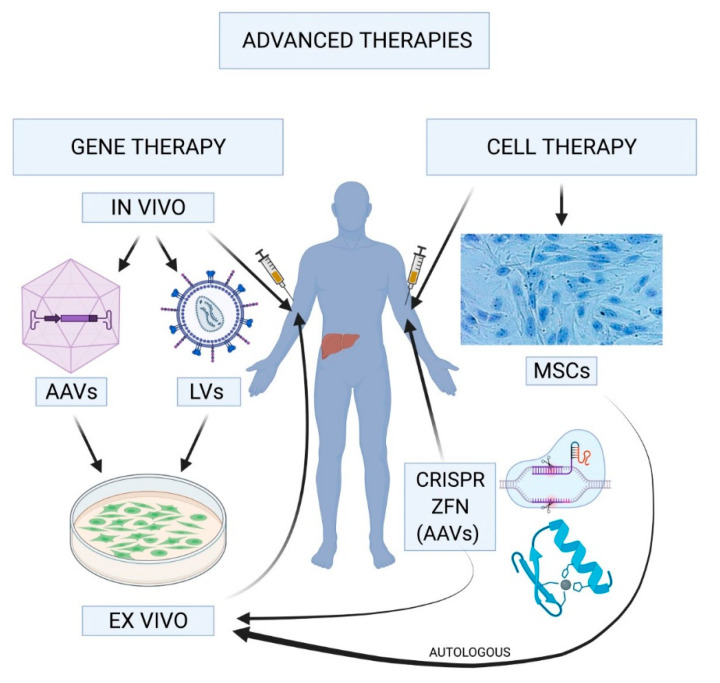
Advanced therapy strategies. In vivo gene therapy where the (typically organ-specific) adeno-associated viral (AAVs) or lentiviral (LVs) vectors carried by the “therapeutic gene” are administered systemically to the patient. In ex vivo gene therapy, autologous or allogenic cells are transfected in vitro and infused back into the patient following their expansion in the culture medium. Cell therapy uses stem cells, or already differentiated cells, transfected or otherwise, to correct a deficiency in the patient’s physiological function. Another alternative is to correct the defective genes responsible for the disease. To do that, several gene-editing tools are available such as CRISPR/Cas9 or ZFNs (zinc finger nucleases). Adeno-associated viral vectors are used to insert guide RNAs and other components necessary to the correction. MSCs, mesenchymal stem cells. (Created with Biorender.com).

Advanced therapies comprise a set of novel and innovative strategies such as cell therapy, gene therapy and regenerative medicine or tissue engineering. They are aimed at conditions or disorders that currently lack a curative treatment of whose treatment requires optimization. According to international medicine agencies, the products used in the context of advanced therapies are drugs for human use that are based on genes, tissues or cells and that offer innovative solutions for the treatment of some diseases [11] (Figure 1).

In hemophilia, protocols based on gene and cell therapy have been shown to hold great potential. Clearly viable cell and gene therapy approaches exist both to address monogenic and polygenic conditions, and to increase the effective duration of therapeutic proteins and boost their level of expression. This can be achieved thanks to the availability of a wide range of varieties and types of target cells and transfection vectors, and because it is now possible to regulate the gene expression and the characteristics of the transgene, and in the end, ensure its safety. Investments in investigating these new therapies are clearly justified as there are multiple chronic and severe conditions for which no treatment is currently available and others for which the existing treatment either results in severe side effects or is tedious and/or inconvenient, making adherence difficult.

Somatic cell therapy is defined as the administration of live autologous, allogenic or xenogenic non-germline cells that have been minimally manipulated or processed ex vivo through propagation, expansion, selection, pharmacological treatment or some other modification of their biological characteristics. The purpose of somatic therapy is to recover a nonexistent or lost function in a living organism. The cells used in cell therapy are generally stem cells equipped with a series of special characteristics, such as being undifferentiated, having the ability to self-renew and to differentiate to different cell lines or to different embryonic layers [12,13,14,15].

Use of induced pluripotent stem cells (iPSCs) in this context is also common as they present with similar characteristics to those of embryonic cells but without the bioethical problems associated with them. IPSCs have allowed significant progress in the realm of HA and HB [16,17].

Of all multipotent stem cells or adult cells, the ones presenting with the highest clinical application potential are the mesenchymal stem cells derived from adipose tissue, bone marrow, umbilical cord and placenta, due to their low immunogenicity, their immunomodulatory effect, and the fact that they do not induce an immunogenic response as they do not express the class II major histocompatibility complex (MHC-II) or T-lymphocyte costimulatory molecules such as CD40L, CD80 or CD86 [18,19].

As far as gene therapy protocols are concerned, these can be defined as the transfer to one or more cells of a DNA fragment that encodes a protein and may be able to cure a certain disease. This simple definition has opened up a whole new dimension in the treatment of a large variety of conditions, ranging from hereditary diseases to cancer and infectious, cardiovascular, hepatic and neurodegenerative conditions [20].

Even if gene therapy protocols are often more effective than those of cell therapy, they are associated with significant difficulties and a greater number of adverse events, mainly related to the transfer vector. An ideal transfer vector should be immunologically inert; highly tissue- and cell-specific; integrative; capable of sustainably producing the transgene and transducing genes to divided or undivided cells; easy to produce at large scale; and capable of maximum transgene loading. However, at the present time, immunogenicity and hepatotoxicity of the transfer vector as well as the problems associated with its integration (insertional mutagenesis) are the most important barriers that must be overcome [21].

Gene therapy protocols can be applied to somatic cells either in vivo, where correction of the gene, or its insertion into the cells, is performed in the body through a vector; or ex vivo, where correction of the gene, or its insertion, is carried out previously in a series of cells harvested from the patient then reimplanted following an expansion and selection process [20,22].

As regards transfection vectors, gene therapy can be classified into viral and non-viral. The higher efficacy of viral vectors as compared with non-viral vectors has made them the better candidates for clinical application [20,23,24]. However, it must be said that retrovirus (RNA virus)-based vectors are practically absent from clinical practice despite their high transduction efficacy and their ability to maintain transgene expression over time. Their drawbacks are related to their high capacity to integrate into the genome and their high insertional mutagenesis. Adenoviral (DNA virus) vectors are non-integrative and are able to efficiently package the “therapeutic” gene but present with a very high immunogenic capacity, which could trigger severe anaphylactic reactions. Adeno-associated virus (AAV) vectors for their part exhibit the greatest potential for clinical use. They are partially integrative vectors associated with a low risk of insertional mutagenesis. However, they are characterized by a high immunogenic response to their capsid and may, in some cases, be hepatotoxic. Lastly, lentiviral vectors (LVs), based on single-stranded RNA lentiviruses, are integrative and not very likely to result in insertional mutagenesis, nor do they induce a significant immune response or produce a hepatotoxic effect. Although, they exhibit the same low packaging ability as other retroviruses, next-generation LVs, which have been enhanced and endowed with protection from phagocytosis, have been shown to achieve optimal FVIII and FIX expression in the liver [25].

Another alternative to therapies consisting of transferring a therapeutic gene is correction of the defective genes causing the condition. Several gene-editing tools are currently in use, such as transcription activator-like effector nucleases (TALENs), zinc finger nucleases (ZFNs) and CRISPR/Cas9 [26,27]. These tools are based on the induction of DNA double-strand breaks and are equipped with a restriction endonuclease in charge of mediating the break and a variable part, which is designed as a complement to the target sequence guiding the endonuclease, where the break is performed. Subsequently, the cell repair system induces the potential correction into the DNA when repairing the break. As regards the CRISPR/Cas9 tool, the variable part is nucleotide-based (RNA) rather than protein-based.

As regards correction by gene editing, a phase 1 study has been initiated [28] to evaluate the long-term safety, tolerability and expression of FIX in patients with severe HB using an AAV vector. SB-FIX is a ZFN-mediated genome-editing therapy administered by means of AAV vectors. SB-FIX is intended to function by placing a corrective copy of the FIX transgene into the genome of the subject’s own hepatocytes, under the control of the highly expressed endogenous albumin locus, thereby providing a permanent, liver-specific expression of FIX.

Gene therapy treatments based on siRNA (small interference RNA) are also being developed. These therapies are intended to modify the translation of messenger RNA, inhibiting or modifying production of the protein without affecting the encoding gene [29].

Recent work in gene therapy aimed at developing a treatment for congenital coagulopathies has instilled renewed hope in patients with HA and HB [4]. Use of different AAV vector genotypes has already yielded concrete, nd highly encouraging, results in clinical trials in terms of the expression levels (moderate phenotype) and expression times (several months) achieved [30]. However, the problems related to the vectors’ immunogenicity and hepatotoxicity remain to be solved.

The first gene therapy clinical studies in coagulopathies, which used retroviral, adenoviral and non-viral (ex vivo) vectors were associated with transient expressions and low levels of the coagulation factor [31,32]. These results led to a change in strategy and to the use of “non integrative” recombinant viruses such as AAVs. This kind of vector derives from a native non-pathogenic and barely immunogenic AAV of the parvovirus family which, given its inability to replicate, requires an auxiliary virus to do so [33].

The DNA sequences transported by AAV vectors are stabilized in an episomal form, so that long-term expression is only possible through delivery into long-living postmitotic cells. The vector’s DNA integrates at a very low rate and is typically lost from replicating cells. Recombinant vectors display tropism for a series of target tissues, such as the liver, which makes them the most commonly used gene therapy viral vectors in hemophilia (90%), followed by in vivo and ex vivo LVs (10%).

Liver-directed gene therapy may transform congenital hemophilia from an incurable disease characterized by a severe phenotype into a moderate or even mild type of hemophilia. However, it is difficult to anticipate what proportion of patients with hemophilia may benefit from the gene therapy, as multiple factors have been described which could restrict its application [34]. For example, very effective treatments such as extended half-life recombinant products, or the new non-replacement-based therapies, are palliative treatments which, when administered prophylactically, are capable of boosting patients’ quality of life. A second hurdle to their implementation is related to the uncertainties around their potential adverse events, mainly derived from transfection vectors (unknown risks and long-term outcomes, persistence of the therapeutic effect, need to readminister the vector). To this should be added the lack of experience of healthcare providers in managing the adverse events derived from the new advanced therapies and their lack of experience in calculating the cost-effectiveness of gene therapy (eligibility criteria, predictability of response, unknown risks, long-term complications). There is also considerable uncertainty as to whether payers will be willing to defray the high upfront costs of the one-and-done cure promised by gene therapy, especially considering that reimbursements are normally made based on real world results. In short, the role of regulatory agencies, payers, patients and healthcare providers will be key in identifying the patient subpopulations most likely to benefit from gene therapy and, therefore, aligning patients’ interests with those of pharmaceutical companies.

Pursuing this strategy will require a definition of variables (serotype/dose of the viral vector, manufacturing techniques) capable of controlling the integration capacity of the different vectors and/or the immune response against the AAV capsid, as well as an evaluation of the patients’ quality of life defined as an improvement in those outcomes that are most relevant for the patients (chronic pain, yearly infusion rate, mental health). Educational programs should be introduced to provide all stakeholders with the information they require about gene therapy as a therapeutic option, including the management of adverse events and the strategies conducive to ensuring the cost-effectiveness of the treatment.

## 3. Adeno-Associated Virus, the Vectors of Choice. Optimization

AAVs are currently the vectors of choice in gene therapy, particularly in the context of congenital coagulopathies. This is due to their good safety profile and their capability for partial integration, which ensures long-term expression of the transgene.

Nonetheless, some serotypes may be hepatotoxic and more immunogenic [35]. For that reason, transient immunosuppressive techniques have been developed to blunt the cell’s immune response against the virus. Paulk et al. [36] reported two AAV serotypes (NP40 and NP59) capable of improving in vivo transduction of human hepatocytes in murine models.

Moreover, pre-existing antibodies against AAVs significantly affect the use of AAV vectors even in the presence of relatively low levels of neutralizing antibodies from natural parvovirus infections. Such capsid-targeted antibodies have been shown to be capable of inhibiting transduction of the intravenously administered vector in animal models and have been associated with limited effectiveness in clinical trials in humans [37]. This is important to determine the primary eligibility of patients for AAV-based gene therapy clinical trials.

Immunosuppression using drugs such as corticosteroids is nowadays necessary to ensure the clinical benefits of AAV-mediated gene transfer. Nonetheless, immunosuppressive drugs are associated with metabolic side effects and with an increased overinfection risk. For this reason, further research is needed to find new, less immunogenic AAV serotypes.

One of the currently most widely accepted hypotheses is that CD8^+^ lymphocytes may play an important role in the subject’s immune response against the vector capsid, eliminating the hepatocytes infected by the virus and triggering an ensuing reduction in the levels of the transgene and of the circulating therapeutic protein. This has prompted the development of new strategies aimed at eradicating the parts of the viral vector responsible for inducing the immune and inflammatory response or providing CD8^+^ lymphocytes with epitopes that enable them to bind to AAVs, thus attenuating the T-lymphocytes’ response [38].

In sum, a great deal of research is still needed into the optimization of AAV vectors, along two different paths. One should be directed at the development of new, less immunogenic serotypes, and the other at the standardization of the clinical dose of vector to be administered. Selecting the first doses of investigative drugs to be administered to humans on the basis of the results of preclinical studies has been, and still is in the context of advanced therapies, an important challenge for translational medicine. Despite the substantial advances made in AAV optimization, significant limitations still exist to the use of preclinical data to guide dose selection in clinical trials.

Tang et al. recently introduced a series of new concepts and parameters aimed at optimizing selection of the AAV vector dose [39]. The novel gene efficiency factor (GEF) concept was developed to describe the efficiency of the in vivo AAV-mediated gene transfer system. It indicates the number of molecules of the therapeutic protein produced per day by each genome of the administered vector (vg). The concept encompasses both the transfection of the virus and the transcription/translation of the transgene required by the gene therapy product to exert its biological effect, and also allows prediction of the most effective dose of the AAV vector for each species. This makes it possible to translate the biological efficacy observed in animal studies to clinical trials in humans, which provides a rational basis to determine the initial dose of the new in vivo virus-mediated gene therapy products.

## 4. Gene Therapy Educational Programs for Patients

At present, patients with hemophilia benefit from palliative treatments providing unprecedented levels of safety and efficacy. These are based on recombinant DNA techniques that offer high levels of safety against emerging pathogens, as well as high efficacy and long half-lives, allowing administration of one single dose every 10 days in the case of FIX and two doses every 10 days in the case of FVIII. This treatment concept makes it difficult for both doctors and patients to switch to a different alternative such as gene therapy, particularly given the uncertainties around its medium- and long-term adverse events. Moreover, it must be remembered that patients with hemophilia tend to be well informed about their disease, the treatments and therapeutic options available and even the management of their disease, as most of them are on home self-treatment. For these reasons, and given the high prevalence of pre-existing AAV antibodies, recruiting patients for gene therapy trials is typically challenging. This usually results in small sample sizes and low statistical significance levels. This highlights the urgency of designing communication and information campaigns aimed at both the clinical staff practicing in the different hemophilia units, and at the patients themselves [40]. The information provided must be objective and realistic, as well as clear and simple, especially when discussing highly specialized concepts related to the fields of genetics and molecular biology. This will definitely contribute to promoting a better understanding of the possibilities, potential risks, benefits and limitations of these new therapeutic protocols.

The information should be conveyed in the form of semi-structured informative sessions where patients should also be asked open-ended questions about their perceptions about the new treatments. In a study by Overbeeke et al. [41], the majority of patients with hemophilia showed a positive attitude toward gene therapy and claimed to be keen to receive this treatment (40%; *n* = 8) o (35%; *n* = 7). The criteria influencing patients’ decisions included the annual bleeding rate, the level of factor, uncertainty about long-term risks, the impact of treatment on their daily lives and the possibility that prophylaxis may be discontinued. Consistent use of specific and generally accepted terms and an optimized narrative (verbal, written and pictorial language) to convey the information could minimize confusion and facilitate the making of informed decisions on the potential offered by gene therapy [42].

Other educational programs geared at patients and healthcare providers include more specific training on matters such as the use of AAVs, whose application in patients with hemophilia is becoming increasingly common. To be effective, these programs require the participation of academically experienced specialists [43]. They are often based on an information handbook that includes information on AAV-mediated gene transfer, the eligibility criteria for participation in clinical trials, the possibility of anticipating the adverse events that could occur in the course of a clinical trial following gene therapy, the parameters that should be monitored during the clinical trial and the long-term effects of the therapy. Handbooks also include easy-to-read infographic materials that physicians can use as visual aids during patient consultations. They also review the basic principles of liver-targeted AAV-mediated gene transfer to facilitate discussions between physicians, or with patients and their families.

## 5. The World Federation of Hemophilia Gene Therapy Registry and the Global Multidisciplinary Consensus Framework on Hemophilia Gene Therapy

Since May 2021, the World Federation of Hemophilia (WFH) has been working on a Gene Therapy Registry [44,45] intended to put together a worldwide database of patients with hemophilia receiving gene therapy. The purpose of the Registry is to gain a comprehensive insight into the conditions under which gene therapy is administered; the expected, unexpected and unknown safety issues; and the duration of the efficacy of therapy over the long term. Against the background of the imminent approval and release of gene therapy drugs addressed to patients with hemophilia, the WFH saw the need to carry out this initiative at a global scale. Every patient will be monitored for 15 years, and any adverse events occurring from the start of administration of therapy will be recorded at 3, 6, 9, 12, 18 and 24 months, and yearly thereafter. Levels of circulating factor and any bleeding episodes will also be recorded from the start. The only inclusion criterion will be to have a diagnosis of HA or HB, with no age distinction, and to have been prescribed gene therapy.

Another important aspect will be reaching a consensus on the use of a multidisciplinary approach to the application of the new gene therapy protocols in hemophilia [46]. Treatment of hemophilia has always been multidisciplinary, with the patient at the center and practitioners from different departments of the hospital working as a team to ensure the delivery of an optimal standard of care [47]. In the case of gene therapy, this is more necessary, and the multidisciplinary team should include practitioners from departments such as molecular biology, clinical pharmacology and virology.

The new gene therapy protocols will require hospital pharmacy departments to revise their storage, handling and reconstitution processes. At the same time, the gastroenterology and hepatology departments will have to monitor the hepatic function of recipients of gene therapy, especially during the first few months of treatment. Physical therapists for their part will have to follow up the condition of the patients’ joints by means of regular physical examinations and musculoskeletal ultrasounds.

In a nutshell, careful multidisciplinary planning will be required [48] before any gene therapy procedures can be applied. The procedure will include an assessment and informed consent phase, dosing, short- and long-term treatment and follow-up. Adverse events and fluctuations in hepatic function will have to be properly managed, and requirements in terms of immunosuppression and availability of resources will have to be met.

## 6. Cost-Effectiveness of Gene Therapy for Hemophilia

Treatment of hemophilia based on plasma-derived products can be very costly, especially if such products are of a recombinant nature [49]. In the United States, the annual cost exceeds USD 300,000 per adult patient. Indirect costs such as the loss of production capacity and the costs arising from the physical disability resulting from hemophilic arthropathy are also considerable. Factor concentrates account for 90% of all direct costs of treating hemophilia.

Certain strategies have been implemented to reduce the economic impact of hemophilia, such as disease management programs and programs to control the price of drugs. Production of longer half-life recombinant coagulation factors capable of reducing dosing frequency has also greatly contributed to reducing costs, with comparable clinical outcomes [50].

As a potential cure for hemophilia, the new advanced therapies are also associated with a high cost, which limits their applicability and raises doubts about their cost-effectiveness and their equitable administration to patients with hemophilia worldwide, ensuring what the WHO has called “the absence of avoidable and remediable differences between groups of people” [51].

Machin et al. [52] carried out a study to evaluate the economics of gene therapy in patients with severe HA as compared with prophylaxis with exogenous FVIII using a Markov state-transition model to estimate the costs and efficacy of treatment of severe HA in the United States. They performed several unidirectional and probabilistic sensitivity analyses to determine the soundness of their results. Over a 10-year period, the total per-patient cost of gene therapy was USD 1.0 million and 8.33 QALYs (quality-adjusted life-years, a parameter that measures the burden of disease, including quality of life and the number of years lived, and evaluates the economic efficiency of medical interventions). The cost of prophylaxis was USD 1.7 million and 6.62 QALYs. The better results obtained by gene therapy, which showed itself to be less costly and more effective than prophylaxis, could mean that gene therapy-based treatment of severe HA is more economically efficient than prophylaxis with FVIII.

In the case of hemophilia B, Bolous et al. [53] compared the potential economic efficiency of AAV-mediated Factor IX-Padua gene therapy in patients with severe HB in the United States with on-demand FIX replacement and primary prophylaxis with standard FIX or extended half-life FIX products. The authors built a Markov microsimulation model using published data as a basis for the transition probabilities between health states and utilities. Over an 18-year period, gene therapy also showed itself to be more economically efficient than on demand or prophylactic treatment in patients with severe HB.

Although further economic studies are required to address this dilemma, it would be misleading to conclude that gene therapy is inherently more costly [54]. A rigorous economic evaluation of the new therapies requires a careful comparison between the costs and benefits of gene therapy and the standard of care without adverse events throughout the patient’s lifetime, including the relevant adjustments for price distortions. This would allow a better estimation of the cost-effectiveness ratio of every treatment.

## 7. Current Gene Therapy Clinical Trials in Hemophilia

An important feature of hemophilia treatment is that circulating factor concentrations need not reach normal concentrations for the patient to obtain a therapeutic effect. Indeed, a slight increase in plasma levels (to more than 1% of the reference value) is enough to reduce the morbidity and mortality risk. This reduces the expectations regarding the target factor correction as it is not necessary to restore circulating factor levels to 100%.

The analysis made in this article is based on some of the clinical trials in progress on HA or HB reported in the ClinicalTrials.gov repository [55] and the European Union Clinical Trials Register [56]. A total of over 40 active gene therapy clinical trials were found on the subject of hemophilia, with an approximate 50/50 split between HA and HB. Table 1 shows the recombinant virus vectors used in the different clinical trials for HA and HB, and Table 2 includes a list of the most significant clinical trials in progress on HA and HB.

One of these clinical trials, sponsored by the Oxford University Hospitals NHS Foundation Trust, will analyze the potential impact of gene therapy on the lives of persons with hemophilia and their family members [57]. It is a multiple-cohort research study to be conducted among diverse groups within the hemophilia community whose lives may have been impacted by gene therapy. The study has been designed to allow patients and their families to tell their own life stories through narrative accounts. The narratives will represent a true sharing of experiences and offer an insight into how these patients and families have coped with hemophilia following the application of the new therapies.

### 7.1. Hemophilia A

AAV-based gene therapies for the treatment of HA are at an earlier stage of development than those for HB. The main problem in the case of FVIII is related to the packaging in the vector as the gene of this protein is much larger (7 kb) and exceeds the packaging capacity of AAVs, which is around 5 kb.

BioMarin Pharmaceutical pioneered the first clinical trial devoted to HA, using hepatic gene transfer with an AAV serotype 5 (AAV5) vector expressing B-domain deleted FVIII (BDD-FVIII-DQ, BMN 270) [58]. Many of the patients participating in the trial (88.8%) experienced a slight increase in their alanine aminotransferase (ALT) levels, which was accompanied by a reduction in the activity of FVIII in one of the subjects. The company has started another two phase 3 trials with vector doses of 4 × 10^13^ and 6 × 10^13^ vg/kg [59,60].

Pasi et al. [61] obtained long-term efficacy and optimal clinical and histological results in 15 adult subjects with severe HA who had received different doses of a single infusion of AAV5-hFVIII-SQ. Three years following the infusion, FVIII expression in patients receiving the highest dose (6 × 10^13^ vg/kg) was 20% of the reference value, the median number of bleeding episodes treated annually was 0 and the use of exogenous FVIII decreased from a median of 138.5 to 0 infusions a year.

BioMarin Pharmaceutical also started a phase 1/2 clinical trial (results still unavailable) to evaluate the effect of administering high doses of the valoctocogene roxaparvovec gene to patients with HA with preexisting antibodies against the AAV5 capsid [62].

### 7.2. Hemophilia B

Generally, the results of the trials carried out so far on the use of AAVs in patients with HA and HB have yielded promising results. A clinical trial sponsored by the St. Jude Children’s Research Hospital in patients with HB, which used a scAAV2/8-LP1-hFIXco vector, was the first to confirm the long-term benefit of gene therapy with this kind of AAV vector [63,64]. The authors evaluated the stability and long-term safety of the expression of the transgene in 10 patients with severe HB, all of whom were infused with a single dose of AAV8 vector. A vector dose-dependent increase in circulating FIX of 1% to 6% was observed over a mean 3-year period, which made it possible to reduce the frequency of prophylactic administration of FIX and, in some cases, even suspend it. All the patients, however, developed capsid-specific antibodies. The main adverse effect encountered was an increase in hepatic enzyme levels (ALT), which was treated with prednisolone.

University College London has started a new phase 1/2 clinical trial focused on HB, using the AAV-F9, FLT180a vector [65].

UniQure Biopharma B.V. recently published the results of a series of clinical trials on HB using an AAV5 vector that uses the same FIX expression cassette as the Jude Children’s Research Hospital (AMT-060) but with the Padua variant of FIX [66,67]. The cassette used in this study was the same wild-type human FIX gene cassette tested previously [68], also with the AAV5 serotype. The study achieved similar steady-state FIX activity levels to a previous study by Nathwani et al. [63], who used an AAV8 vector. However, the highest vector dose used in the UniQure Biopharma BV study was higher than the AAV8 vector dose used by Nathwani et al. The authors concluded that pseudotyped scAAV5 vectors ought to be effective in the treatment of individuals with preexisting immunity to AAV5 [69,70].

In a recent clinical trial, Konkle et al. [71] analyzed safety, pharmacokinetic profile, FIX activity and immune response in patients undergoing a gene therapy protocol based on the AAV virus serotype 8 (AskBio009) [72]. Eight adult males were administered different doses of the gene therapy product BAX 335 IV (2.0 × 10^11^; 1.0 × 10^12^; or 3.0 × 10^12^ vg/kg), with three patients (37.5%) developing severe complications, all of them considered to be unrelated to BAX 335. No clinical thromboses, inhibitors or immunity reactions were observed against the Padua variant of FIX.

### 7.3. Padua Variants of Factor IX. An Exciting Alternative for Gene Therapy in HB

Although the results of AAV-based gene therapy using FIX as a transgene have been encouraging in the context of HB, increases in hepatotoxicity resulting from the loss of expression of the transgene are not uncommon, possibly due to adaptative or innate immune responses against the vector’s capsid.

One alternative to reduce hepatotoxicity is to reduce the vector dose, but this usually results in a decreased expression efficacy. Another possibility is to modify the transgene itself to boost the efficiency of the coagulation factor once it has expressed itself. In this regard, Lombardi et al. [73] recently applied protein fusion engineering to design a FIX-HSA (human serum albumin) variant that lengthens the half-life of the FIX molecule. This combination could be used as a transgene in gene therapy protocols in the context of HB. Yet another alternative would be to use some variant of the protein endowed with greater clotting capacity, such as the Padua variant of FIX [74]. Juvenile thrombophilia is associated with the presence of the Padua variant of FIX, which is characterized by the substitution of leucine by arginine at position 338 (R338L) (FIX-R338L). Plasma concentrations of the FIX-R338L protein are normal but its clotting activity is approximately eight times higher than normal. In vitro, the specific activity of recombinant FX-R338L is 5–10 times higher than that of wild-type recombinant FIX (FIX-WT). Substitution of R338L leads to a gain-of-function mutation, which in turn results in a hyperfunctional FIX.

The clinical and preclinical experience with FIX-R338L gene therapy in the realm of HB has not provided evidence of a higher thrombogenic or immunogenic risk [68,75,76]. However, as the mechanism underlying R338L’s clotting hyperactivity remains unknown, the potential adverse events associated with a random coagulation pattern and the possibility of developing thrombotic complications cannot be ascertained. Samelson-Jones et al. [77] showed that the high specific activity of FIX-R338L requires FVIIIa as a cofactor. The molecular regulation of the activation, inactivation and FVIIIa-dependence of FIX-R338L and FIX-WT are similar, but the FVIIIa-dependent hyperactivity of FIX-R338L is the result of a faster activation rate of FX. These findings helped dispel the fears associated with the unregulated clotting pattern of FIX-R338L and support its use in gene therapy protocols addressed at HB. Another study by Samelson-Jones et al. in dogs with HB [78] confirmed an increased specific in vivo activity of FIX-R338L as compared with FIX-WT following gene therapy with AAV6.

At present, Pfizer is conducting a clinical trial with the Padua variant of FIX [79,80]. The study is intended to evaluate the efficacy and safety of PF-06838435 (rAAV-SPARK100-HFIX-PADUA) in male adult subjects with moderate or severe HB, with circulating FIX concentrations <2%.

Nair et al. [81,82] tested a new modification of the Padua variant of FIX. In clinical trials with FIX-R338L, they found that some patients showed an increase in liver transaminase levels, which was correlated with the loss of expression of FIX, even in immunosuppressed subjects. These results underscore the urgency to look for new, more effective variants that may allow a reduction in the vector dose and, therefore, prevent potential adverse events. The study described how new variants could be generated.

Thus, dalcinonacog alpha, a new variant of R338L-Padua also known as CB 2679d-GT, containing three amino acid substitutions (R318Y, R338E, T343R), was shown to be more potent than R338L-Padua following AAV-based gene therapy in hemophilic mice. A significant (five to eight-fold) reduction in bleeding time and of the total amount of blood lost (approximately four-fold) was obtained compared to R338L-Padua, which allows a faster and more robust hemostatic correction. FIX expression remained stable for at least 20 weeks, and no significant increases in immunogenicity were observed. This novel gene therapy study demonstrated the superiority of CB 2679d-GT, highlighting its potential to obtain higher levels of FIX activity and superior hemostatic efficiency following AAV-based gene therapy in patients with HB.

## 8. Discussion

The current treatment of choice for hemophilia is fundamentally based on intravenous prophylactic replacement of the deficient factor by exogenous recombinant factors, characterized by their longer half-lives, their robust safety profile against emerging pathogens and their high efficacy. This has allowed patients to lead normal lives and resume their social and occupational activities.

The drawbacks of this treatment include its high administration frequency and inconvenient route of administration, as well as the development of inhibitors by up to 30% of patients against the infused coagulation factors.

Advanced (gene and cell) therapies could provide a “cure” for many hereditary conditions such as hemophilia. Gene and cell therapy protocols are appropriate for monogenic and polygenic diseases and should allow expression of the deficient factor over the long term and the maintenance of steady-state plasma concentrations. Protocols are currently highly variable on account of the wide range of target cell types and gene transfection vectors available and the possibility of modifying the transgene to boost its expression efficacy. The investment and dedication required by these procedures is fully justified as many of the conditions they are meant to treat are chronic and/or severe and either lack a curative treatment or are associated with tedious or inconvenient treatments or therapies, leading to significant side effects which hinder patient adherence.

Although gene therapy protocols are in general more efficient than cell therapy ones, they may be associated with a higher incidence of adverse events, derived mainly from the viral transfection vector used. The vector’s immunogenicity and hepatotoxicity and the problems arising from its integration (insertional mutagenesis) are the main hurdles to overcome. The choice between a viral or non-viral vector, and between the different kinds of viral vectors available, must be based on a compromise between the phenotypic features of the disease and the therapeutic targets pursued. Highly effective vectors usually result in a faster and more efficient translation to clinical practice. AAV vectors are currently the ones presenting with the highest potential for clinical use given their partial integration capacity and their low insertional mutagenesis risk. It must, however, be remembered that they may lead to an intense immunogenic response and, in some cases, to hepatotoxicity.

One alternative to therapeutic gene-based gene therapy is correction of the defective genes causing the condition through a wide range of gene-editing tools (TALENs, ZFNs and CRISPR/Cas9).

The advances made in the last few years by gene therapy for the treatment of congenital coagulopathies have caused an unprecedented upheaval, specifically in the treatment of patients with HA and HB. Indeed, the use of AAV vectors has provided extraordinarily promising results in terms of the clotting factors’ concentrations and expression times. However, further work will be necessary to address the side effects related to the immunogenicity and hepatotoxicity of these therapies, which usually make it necessary to prescribe concomitant administration of immunosuppressive corticosteroids.

As a result, switching the therapeutic strategy from the current optimized and safe standard-of-care treatment based on recombinant products to a gene therapy strategy is not easy, especially considering the uncertainties around the medium- and long-term effects of gene therapy protocols, the lack of experience with these new procedures and the potentially unfavorable cost-effectiveness ratio associated with them. For these reasons, further research must be conducted with a view to optimizing AAV vectors, and should come up with less immunogenic and hepatotoxic serotypes. Concomitant use of corticosteroid-based immunosuppressants should be avoided given their metabolic side effects and the fact that they may increase the risk of overinfection [83].

Providing patients with hemophilia with adequate information is now more necessary than ever. Information must be objective and realistic, but at the same time clear and easy to understand, especially when dealing with highly specialized concepts related to genetics and molecular biology. This will contribute to avoiding unrealistic patient expectations and promoting a deeper understanding of the potential risks, benefits and limitations of the new therapeutic protocols. It will also be necessary to keep a detailed record of patients with hemophilia receiving gene therapy across the world in order to track expected, unexpected and unknown safety issues and obtain a better insight into the maintenance of the protocols’ efficacy in the long term. Appropriate multidisciplinary planning will also be necessary at the different stages of the procedure, including evaluation, informed consent, dosing and short- and long-term treatment and follow-up.

Although treatments based on advanced therapies are costly, several economic forecasting studies have demonstrated that they could become less costly than the currently used regimens. At the same time, every effort should be made to address the current inequalities in access to treatment by promoting social access and ensuring that patients receive the treatments they need regardless of geographical or economic factors.

As regards the efficacy of gene therapy protocols in the context of hemophilia, expectations can be relaxed to some extent as even a small increase in circulating factor concentrations to more than 1% of the reference value, which corresponds to a severe phenotype, would result in a significant reduction in morbidity and mortality.

In the case of HA, use of AAV vectors is at an earlier stage of development than in HB. Solid foundations should be laid to provide an effective solution to the issue of the FVIII gene that is too large to be packaged into a given vector, using a gene deleted from the B domain of FVIII. This is not an issue in HB as the FIX gene is smaller than the FVIII gene. The problem for both HA and HB remains the immunogenicity and hepatotoxicity of the AAV vector, which are related to the loss of expression of the transgene. Although one alternative is concomitant treatment with corticosteroid immunosuppressants, these drugs should be avoided given their poor side effect profile [83]. Reduction in the vector dose should also be avoided as it is often associated with a less efficient expression of the protein. A third alternative is to modify the transgene to boost the efficacy of the clotting factor once it has been expressed. This last possibility, together with the use of variants with a higher clotting ability, such as the Padua variant of FIX (factor IX-R338L), currently represents an exciting alternative for HB patients. The greater coagulating activity of the protein expressed from a transgene variant makes it possible to reduce the vector dose without affecting expression efficacy, thus minimizing the risk of adverse events.

More recently, a subvariant of FIX-R338L (CB 2679d-GT) that contains three amino acid substitutions (R318Y, R338E, T343R) has been shown to be more effective in reducing bleeding times and total blood loss in gene therapy protocols with AAV vectors. FIX expression persisted for at least 20 weeks and immunogenicity remained unchanged.

Gene therapy could cause a great upheaval in the treatment of patients with hemophilia and other congenital diseases. Although more work is needed to increase treatment efficacy and reduce adverse events such as immunogenicity and hepatotoxicity, these protocols could allow curation of the disease, thereby increasing the patients’ quality of life, and a reduction in both direct and indirect costs throughout the lifetime of patients suffering from chronic lifelong diseases [54].

Protocols based on gene therapy have gone from being potentially applicable ideas to becoming tangible realities [84], and they are bound to herald a new era where analyzing the specific phenotype of a monogenic hereditary disease such as hemophilia may not be needed anymore [85]. Gene therapy is indeed a tangible reality and the first gene therapy-based products targeted at hemophilia will be available in the short term [86].

We are already well into the 21st century, an era in which precision and personalized medicine [87] are gaining huge momentum in the realm of pharmacology. The rise of gene therapy is a good example of that. Nevertheless, this has not happened without significant controversy between its advocates and its critics regarding its promises, limits, possibilities and opportunities. Although huge strides have been made in our understanding of the molecular mechanisms behind diseases and in the development of drugs that have had a hugely positive impact on the treatment of some types of cancer, many of the promises of gene therapy remain unfulfilled for other diseases as a result of the hurdles to the therapy’s widespread use. The high cost of the new therapies could exacerbate existing inequalities and become a problem for the sustainability of healthcare systems, particularly in low- and medium-income countries. For that reason, it is of the essence for the international community to implement the advances made in an equitable way so as to reduce healthcare disparities [88].

The ultimate goal of the treatment of hemophilia should be a “functional cure”, but also “healthcare equity”. A “functional cure” means that patients should live as normal a life as possible, with minimum joint impairment, an absence of spontaneous bleeds, “normal” mobility, an ability to overcome minor trauma without requiring surgery or any other intervention and normal hemostasis. At the same time, the principle of healthcare equity should be upheld. By eliminating the need for (exogenous) plasma-derived concentrates or recombinant coagulation factors, gene therapy can make healthcare equality possible [89].

## Figures and Tables

**Table 1:** Recombinant viral vectors used in the different clinical trials currently in progress on hemophilia A (HA) and hemophilia B (HB).

Recombinant Vector	Type of Hemophilia
Adeno-associated virus serotype 5 vector containing a variant of B-domain deleted human FVIII ^a^ known as BMN 270)	HA
Recombinant adeno-associated virus serotype 6 vector encoding B-domain deleted human FVIII (known as SB-525)	HA
Adeno-associated virus serotype 8 vector containing a functional copy of the codon-optimized FVIII’s cDNA encoding B-domain deleted FVIII	HA
Recombinant adeno-associated virus vector containing a bioengineered capsid and a codon-optimized expression cassette to drive the expression of the SQ form of a B-domain deleted human FVIII (known as SPK-8011)	HA
Non-replicating adeno-associated virus serotype 2 vector expressing the Padua variant (R338L) of human FIX ^b^, under the control of the liver-specific apolipoproteín E/alpha1-antitrypsin (hAAT) enhancer	HB
Adeno-associated virus serotype rh10 vector containing the human FIX gene	HB
Recombinant adeno-associated virus serotype 3 vector containing a codon-optimized expression cassette encoding a variant of human FIX known as FLT180a	HB
Recombinant adeno-associated virus vector containing codon-optimized FIX-Padua (known as AMT-061)	HB
Lentiviral vector encoding human FVIII or FIX	HA and HB

^a^ FVIII, factor VIII. ^b^ FIX, factor IX.

**Table 2:** Main clinical trials underway in the field of hemophilia A (HA) and hemophilia B (HB).

Title	NCT Number	Intervention	Sponsor
Gene therapy study in patients with severe HA with antibodies against AAV5 ^a^	NCT03520712	Adeno-associated virus serotype 5 vector containing a B-deleted variant of FVIII ^b^ (valoctocogene roxaparvovec)	BioMarin Pharmaceutical
Gene therapy study in patients with severe HA	NCT02576795	Valoctocogene roxaparvovec-BMN 270	BioMarin Pharmaceutical
Study evaluating the efficacy and safety of valoctocogene roxaparvovec in patients with HA	NCT03370913	Valoctocogene roxaparvovec	BioMarin Pharmaceutical
Study evaluating the efficacy and safety of volactocogene roxaparvovec combined with prophylactic administration of corticosteroids in HA	NCT04323098	Valoctocogene roxaparvovec	BioMarin Pharmaceutical
Single-arm study evaluating the efficacy and safety of a dose of 4 × 10^13^ vg/kg of valoctocogene roxaparvovec in patients with HA	NCT03391974	Valoctocogene roxaparvovec	BioMarin Pharmaceutical
Gene therapy for HA	NCT03001830	New adeno-associated virus serotype 8 capsid vector pseudotype encoding FVIII-V3 (AAV2/8-HLP-FVIII-V3)	University College, London/Medical Research Council
Safety and dose-escalation study of an adeno-associated virus vector used as gene therapy in HA	NCT03370172	Adeno-associated virus serotype 8 (AAV8) expressing FVIII Factor VIII (BDD-FVIII) (BAX 888)	Baxalta, now part of Shire
Study evaluating the efficacy and safety of PF-07055480 in adults with moderate or severe HA	NCT04370054	Recombinant AAV2/6 encoding B-domain deleted FVIII cDNA	UniQure Biopharma BV
Gene therapy study of recombinant AAV2/6 with the FVIII gene (SB-525) in patients with severe HA	NCT03061201	Recombinant adeno-associated virus serotype 6 (AAV6) encoding B-domain deleted human FVIII cDNA	Pfizer
Study of AAV5-hFIX ^c^ in patients with moderate or severe HB	NCT02396342	AAV5 containing the human FIX gene (AAV5-hFIX)	UniQure Biopharma BV
Dose confirmation trial of AAV5-hFIXco-Padua	NCT03489291	Recombinant adeno-associated virus serotype 5 (AAV5) vector containing the Padua variant of a codon-optimized complementary human FIX under the control of a liver-specific promoter (AAV5-hFIXco-Padua, AMT-061)	UniQure Biopharma BV
HOPE-B: Study of AMT-061 in patients with moderate or severe HB	NCT03569891	AAV5-hFIXco-Padua, AMT-061	UniQure Biopharma BV
Single ascending dose of adeno-associated virus serotype 8 of FIX in adults with HB	NCT01687608	Adeno-associated virus serotype 8 for FIX gene therapy (AskBio009)	Baxalta now part of Shire
Phase 1–2 study of SHP648, an adeno-associated virus vector for gene therapy in patients with HB	NCT04394286	Adeno-associated virus serotype 8 (AAV8) vector expressing FIX Padua (SHP648)	Baxalta now part of Shire
Dose-escalation study of a complementary adeno-associated virus gene therapy vector in patients with HB	NCT00979238	Self-complementary adeno-associated virus serotype 8 (AAV8) vector expressing a transgene of codon-optimized FIX (scAAV2/8-LP1-hFIXco)	St. Jude Children’s Research Hospital/National Heart, Lung, and Blood Institute (NHLBI)/Hemophilia of Georgia, Inc./Children’s Hospital of Philadelphia/University College, London
Long-term safety and efficacy study of SPK-9001 in patients with HB	NCT03307980	Non-replicating adeno-associated virus serotype 2 (AAV2) vector expressing the Padua variant (R338L) of human FIX under the control of the liver-specific apolipoproteín E (Apo E) enhancer (PF-06838435/fidanacogene elaparvovec)	Pfizer
Study evaluating the efficacy and safety of gene therapy with PF-06838435 in adult males with moderate or severe HB	NCT03861273	Fidanacogene elaparvovec	Pfizer
Lentiviral FVIII Gene Therapy	NCT03217032	Lentiviral factor VIII gene. Modified autologous stem cells (YUVAGT-F801)	Shenzhen Geno-Immune Medical Institute
Lentiviral FIX Gene Therapy	NCT03961243	Lentiviral factor IX gene. Modified autologous stem cells (YUVA-GT-F901)	Shenzhen Geno-Immune Medical Institute

^a^ AAV, adeno-associated viral vector. ^b^ FVIII, factor VIII. ^c^ FIX, factor IX.

## References

[1] Castaman G., Matino D. (2019). Hemophilia A and B: Molecular and Clinical Similarities and Differences. Haematologica.

[2] Mannucci P.M., Franchini M. (2013). Is Haemophilia B Less Severe than Haemophilia A?. Haemophilia.

[3] Trinchero A., Sholzberg M., Matino D. (2020). The Evolution of Hemophilia Care: Clinical and Laboratory Advances, Opportunities, and Challenges. Hämostaseologie.

[4] Tomeo F., Mariz S., Brunetta A.L., Stoyanova-Beninska V., Penttila K., Magrelli A. (2021). Haemophilia, State of the Art and New Therapeutic Opportunities, a Regulatory Perspective. Br. J. Clin. Pharmacol..

[5] Shima M., Hanabusa H., Taki M., Matsushita T., Sato T., Fukutake K., Fukazawa N., Yoneyama K., Yoshida H., Nogami K. (2016). Factor VIII–Mimetic Function of Humanized Bispecific Antibody in Hemophilia A. N. Engl. J. Med..

[6] Shetty S., Vora S., Kulkarni B., Mota L., Vijapurkar M., Quadros L., Ghosh K. (2007). Contribution of Natural Anticoagulant and Fibrinolytic Factors in Modulating the Clinical Severity of Haemophilia Patients. Br. J. Haematol..

[7] Pasi K.J., Rangarajan S., Georgiev P., Mant T., Creagh M.D., Lissitchkov T., Bevan D., Austin S., Hay C.R., Hegemann I. (2017). Targeting of Antithrombin in Hemophilia A or B with RNAi Therapy. N. Engl. J. Med..

[8] Machin N., Ragni M.V. (2018). An Investigational RNAi Therapeutic Targeting Antithrombin for the Treatment of Hemophilia A and B. J. Blood Med..

[9] Dockal M., Hartmann R., Fries M., Thomassen M.C.L.G.D., Heinzmann A., Ehrlich H., Rosing J., Osterkamp F., Polakowski T., Reineke U. (2014). Small Peptides Blocking Inhibition of Factor Xa and Tissue Factor-Factor VIIa by Tissue Factor Pathway Inhibitor (TFPI). J. Biol. Chem..

[10] Weyand A.C., Grzegorski S.J., Rost M.S., Lavik K.I., Ferguson A.C., Menegatti M., Richter C.E., Asselta R., Duga S., Peyvandi F. (2019). Analysis of Factor V in Zebrafish Demonstrates Minimal Levels Needed for Early Hemostasis. Blood Adv..

[11] Shukla V., Seoane-Vazquez E., Fawaz S., Brown L., Rodriguez-Monguio R. (2019). The Landscape of Cellular and Gene Therapy Products: Authorization, Discontinuations, and Cost. Hum. Gene Ther. Clin. Dev..

[12] Khan W.S., Hardingham T.E. (2012). Mesenchymal Stem Cells, Sources of Cells and Differentiation Potential. J. Stem Cells.

[13] Kolios G., Moodley Y. (2013). Introduction to Stem Cells and Regenerative Medicine. Respiration.

[14] Bacakova L., Zarubova J., Travnickova M., Musilkova J., Pajorova J., Slepicka P., Kasalkova N.S., Svorcik V., Kolska Z., Motarjemi H. (2018). Stem Cells: Their Source, Potency and Use in Regenerative Therapies with Focus on Adipose-Derived Stem Cells—A Review. Biotechnol. Adv..

[15] Ma Q., Liao J., Cai X. (2018). Different Sources of Stem Cells and Their Application in Cartilage Tissue Engineering. Curr. Stem Cell Res. Ther..

[16] Olgasi C., Talmon M., Merlin S., Cucci A., Richaud-Patin Y., Ranaldo G., Colangelo D., Di Scipio F., Berta G.N., Borsotti C. (2018). Patient-Specific IPSC-Derived Endothelial Cells Provide Long-Term Phenotypic Correction of Hemophilia A. Stem Cell Rep..

[17] He Q., Wang H.-H., Cheng T., Yuan W.-P., Ma Y.-P., Jiang Y.-P., Ren Z.-H. (2017). Genetic Correction and Hepatic Differentiation of Hemophilia B-Specific Human Induced Pluripotent Stem Cells. Chin. Med. Sci. J..

[18] Zhuang W.-Z., Lin Y.-H., Su L.-J., Wu M.-S., Jeng H.-Y., Chang H.-C., Huang Y.-H., Ling T.-Y. (2021). Mesenchymal Stem/Stromal Cell-Based Therapy: Mechanism, Systemic Safety and Biodistribution for Precision Clinical Applications. J. Biomed. Sci..

[19] Wu X., Jiang J., Gu Z., Zhang J., Chen Y., Liu X. (2020). Mesenchymal Stromal Cell Therapies: Immunomodulatory Properties and Clinical Progress. Stem Cell Res. Ther..

[20] High K.A., Roncarolo M.G. (2019). Gene Therapy. N. Engl. J. Med..

[21] Bolt M.W., Brady J.T., Whiteley L.O., Khan K.N. (2021). Development Challenges Associated with RAAV-Based Gene Therapies. J. Toxicol. Sci..

[22] Anguela X.M., High K.A. (2019). Entering the Modern Era of Gene Therapy. Annu. Rev. Med..

[23] Wagner H.J., Weber W., Fussenegger M. (2021). Synthetic Biology: Emerging Concepts to Design and Advance Adeno-Associated Viral Vectors for Gene Therapy. Adv. Sci..

[24] Toon K., Bentley E.M., Mattiuzzo G. (2021). More than Just Gene Therapy Vectors: Lentiviral Vector Pseudotypes for Serological Investigation. Viruses.

[25] Cantore A., Naldini L. (2021). WFH State-of-the-art Paper 2020: In Vivo Lentiviral Vector Gene Therapy for Haemophilia. Haemophilia.

[26] Barman H.K., Rasal K.D., Chakrapani V., Ninawe A.S., Vengayil D.T., Asrafuzzaman S., Sundaray J.K., Jayasankar P. (2017). Gene Editing Tools: State-of-the-Art and the Road Ahead for the Model and Non-Model Fishes. Transgenic Res..

[27] Gupta S.K., Shukla P. (2017). Gene Editing for Cell Engineering: Trends and Applications. Crit. Rev. Biotechnol..

[28] U.S. National Library of Medicine Ascending Dose Study of Genome Editing by Zinc Finger Nuclease Therapeutic SB-FIX in Subjects with Severe Hemophilia B. ClinicalTrials.gov Identifier: NCT02695160. NCT02695160.

[29] Adachi H., Hengesbach M., Yu Y.-T., Morais P. (2021). From Antisense RNA to RNA Modification: Therapeutic Potential of RNA-Based Technologies. Biomedicines.

[30] Arruda V.R., Weber J., Samelson-Jones B.J. (2021). Gene Therapy for Inherited Bleeding Disorders. Semin. Thromb. Hemost..

[31] Swystun L.L., Lillicrap D. (2016). Gene Therapy for Coagulation Disorders. Circ. Res..

[32] Chapin J.C., Monahan P.E. (2018). Gene Therapy for Hemophilia: Progress to Date. BioDrugs.

[33] Atchison R.W., Casto B.C., Hammon W. (1965). McD. Adenovirus-Associated Defective Virus Particles. Science.

[34] Spadarella G., Di Minno A., Brunetti-Pierri N., Mahlangu J., Di Minno G. (2021). The Evolving Landscape of Gene Therapy for Congenital Haemophilia: An Unprecedented, Problematic but Promising Opportunity for Worldwide Clinical Studies. Blood Rev..

[35] Verdera H.C., Kuranda K., Mingozzi F. (2020). AAV Vector Immunogenicity in Humans: A Long Journey to Successful Gene Transfer. Mol. Ther..

[36] Paulk N.K., Pekrun K., Zhu E., Nygaard S., Li B., Xu J., Chu K., Leborgne C., Dane A.P., Haft A. (2018). Bioengineered AAV Capsids with Combined High Human Liver Transduction In Vivo and Unique Humoral Seroreactivity. Mol. Ther..

[37] Daniel H.D.-J., Kumar S., Kannangai R., Lakshmi K.M., Agbandje-Mckenna M., Coleman K., Srivastava A., Srivastava A., Abraham A.M. (2021). Prevalence of Adeno-Associated Virus 3 Capsid Binding and Neutralizing Antibodies in Healthy and Hemophilia B Individuals from India. Hum. Gene Ther..

[38] Ertl H.C.J. (2021). T Cell-Mediated Immune Responses to AAV and AAV Vectors. Front. Immunol..

[39] Tang F., Wong H., Ng C.M. (2021). Rational Clinical Dose Selection of Adeno-Associated Virus-Mediated Gene Therapy Based on Allometric Principles. Clin. Pharmacol. Ther..

[40] Miesbach W., O’Mahony B., Key N.S., Makris M. (2019). How to Discuss Gene Therapy for Haemophilia? A Patient and Physician Perspective. Haemophilia.

[41] Overbeeke E., Michelsen S., Hauber B., Peerlinck K., Hermans C., Lambert C., Goldman M., Simoens S., Huys I. (2021). Patient Perspectives Regarding Gene Therapy in Haemophilia: Interviews from the PAVING Study. Haemophilia.

[42] Hart D.P., Branchford B.R., Hendry S., Ledniczky R., Sidonio R.F., Négrier C., Kim M., Rice M., Minshall M., Arcé C. (2021). Optimizing Language for Effective Communication of Gene Therapy Concepts with Hemophilia Patients: A Qualitative Study. Orphanet J. Rare Dis..

[43] Sidonio R.F., Pipe S.W., Callaghan M.U., Valentino L.A., Monahan P.E., Croteau S.E. (2021). Discussing Investigational AAV Gene Therapy with Hemophilia Patients: A Guide. Blood Rev..

[44] Konkle B.A., Coffin D., Pierce G.F., Clark C., George L., Iorio A., Mahlangu J., Naccache M., O’Mahony B., Peyvandi F. (2020). World Federation of Hemophilia Gene Therapy Registry. Haemophilia.

[45] U.S. National Library of Medicine The World Federation of Hemophilia Gene Therapy Registry (WFH GTR). ClinicalTrials.gov Identifier: NCT04883710. NCT04883710.

[46] Pierce G.F., Pasi K.J., Coffin D., Kaczmarek R., Lillicrap D., Mahlangu J., Rottellini D., Sannié T., Srivastava A., VandenDriessche T. (2020). Towards a Global Multidisciplinary Consensus Framework on Haemophilia Gene Therapy: Report of the 2nd World Federation of Haemophilia Gene Therapy Round Table. Haemophilia.

[47] Drayton Jackson M., Bartman T., McGinniss J., Widener P., Dunn A.L. (2019). Optimizing Patient Flow in a Multidisciplinary Haemophilia Clinic Using Quality Improvement Methodology. Haemophilia.

[48] Miesbach W., Pasi K.J., Pipe S.W., Hermans C., O’Mahony B., Guelcher C., Steiner B., Skinner M.W. (2021). Evolution of Haemophilia Integrated Care in the Era of Gene Therapy: Treatment Centre’s Readiness in United States and EU. Haemophilia.

[49] Rodriguez-Merchan E.C. (2020). The Cost of Hemophilia Treatment: The Importance of Minimizing It without Detriment to Its Quality. Expert Rev. Hematol..

[50] Mannucci P.M., Cortesi P.A., Di Minno M.N.D., Sanò M., Mantovani L.G., Di Minno G. (2021). Comparative Analysis of the Pivotal Studies of Extended Half-life Recombinant FVIII Products for Treatment of Haemophilia A. Haemophilia.

[51] (2020). Equity vs. Equality: What’s the Difference? World Health Organization. Milken Institute School of Public Health. https://onlinepublichealth.gwu.edu/resources/equity-vs-equality/.

[52] Machin N., Ragni M.V., Smith K.J. (2018). Gene Therapy in Hemophilia A: A Cost-Effectiveness Analysis. Blood Adv..

[53] Bolous N.S., Chen Y., Wang H., Davidoff A.M., Devidas M., Jacobs T.W., Meagher M.M., Nathwani A.C., Neufeld E.J., Piras B.A. (2021). The Cost-Effectiveness of Gene Therapy for Severe Hemophilia B: Microsimulation Study from the United States Perspective. Blood.

[54] Garrison L.P., Jiao B., Dabbous O. (2021). Gene Therapy May Not Be as Expensive as People Think: Challenges in Assessing the Value of Single and Short-Term Therapies. J. Manag. Care Spec. Pharm..

[55] ClinicalTrials.gov. https://clinicaltrials.gov/ct2/home.

[56] EU Clinical Trials Register. https://www.clinicaltrialsregister.eu/.

[57] U.S. National Library of Medicine An Exploration of the Impact of Gene Therapy on the Lives of People with Haemophilia and Their Families. ClinicalTrials.gov Identifier: NCT04723680. NCT04723680.

[58] U.S. National Library of Medicine Gene Therapy Study in Severe Haemophilia a Patients (270-201). ClinicalTrials.gov Identifier: NCT02576795. NCT02576795.

[59] U.S. National Library of Medicine Single-Arm Study to Evaluate the Efficacy and Safety of Valoctocogene Roxaparvovec in Hemophilia a Patients at a Dose of 4E13 vg/kg (BMN270-302). ClinicalTrials.gov Identifier: NCT03392974. NCT03392974.

[60] U.S. National Library of Medicine Single-Arm Study to Evaluate the Efficacy and Safety of Valoctocogene Roxaparvovec in Hemophilia a Patients (BMN 270-301). ClinicalTrials.gov Identifier: NCT03370913. NCT03370913.

[61] Pasi K.J., Rangarajan S., Mitchell N., Lester W., Symington E., Madan B., Laffan M., Russell C.B., Li M., Pierce G.F. (2020). Multiyear Follow-up of AAV5-HFVIII-SQ Gene Therapy for Hemophilia A. N. Engl. J. Med..

[62] U.S. National Library of Medicine Gene Therapy Study in Severe Hemophilia a Patients with Antibodies against AAV5 (270-203). ClinicalTrials.gov Identifier: NCT03520712. NCT03520712.

[63] Nathwani A.C., Reiss U.M., Tuddenham E.G.D., Rosales C., Chowdary P., McIntosh J., Della Peruta M., Lheriteau E., Patel N., Raj D. (2014). Long-Term Safety and Efficacy of Factor IX Gene Therapy in Hemophilia B. N. Engl. J. Med..

[64] U.S. National Library of Medicine Dose-Escalation Study of a Self Complementary Adeno-Associated Viral Vector for Gene Transfer in Hemophilia B. ClinicalTrials.gov Identifier: NCT00979238. NCT00979238.

[65] U.S. National Library of Medicine A Factor IX Gene Therapy Study (FIX-GT) (FIX-GT). ClinicalTrials.gov Identifier: NCT03369444. NCT03369444.

[66] U.S. National Library of Medicine Trial of AAV5-hFIX in Severe or Moderately Severe Hemophilia B. ClinicalTrials.gov Identifier: NCT02396342. NCT02396342.

[67] U.S. National Library of Medicine Dose Confirmation Trial of AAV5-hFIXco-Padua. ClinicalTrials.gov Identifier: NCT03489291. NCT03489291.

[68] Miesbach W., Meijer K., Coppens M., Kampmann P., Klamroth R., Schutgens R., Tangelder M., Castaman G., Schwäble J., Bonig H. (2018). Gene Therapy with Adeno-Associated Virus Vector 5–Human Factor IX in Adults with Hemophilia B. Blood.

[69] Von Drygalski A., Giermasz A., Castaman G., Key N.S., Lattimore S., Leebeek F.W.G., Miesbach W., Recht M., Long A., Gut R. (2019). Etranacogene Dezaparvovec (AMT-061 Phase 2b): Normal/near Normal FIX Activity and Bleed Cessation in Hemophilia B. Blood Adv..

[70] U.S. National Library of Medicine HOPE-B: Trial of AMT-061 in Severe or Moderately Severe Hemophilia B Patients. ClinicalTrials.gov Identifier: NCT03569891. NCT03569891.

[71] Konkle B.A., Walsh C.E., Escobar M.A., Josephson N.C., Young G., von Drygalski A., McPhee S.W.J., Samulski R.J., Bilic I., de la Rosa M. (2021). BAX 335 Hemophilia B Gene Therapy Clinical Trial Results: Potential Impact of CpG Sequences on Gene Expression. Blood.

[72] U.S. National Library of Medicine HOPE-B: Open-Label Single Ascending Dose of Adeno-associated Virus Serotype 8 Factor IX Gene Therapy in Adults with Hemophilia B. ClinicalTrials.gov Identifier: NCT01687608. NCT01687608.

[73] Lombardi S., Aaen K.H., Nilsen J., Ferrarese M., Gjølberg T.T., Bernardi F., Pinotti M., Andersen J.T., Branchini A. (2021). Fusion of Engineered Albumin with Factor IX Padua Extends Half-life and Improves Coagulant Activity. Br. J. Haematol..

[74] Simioni P., Tormene D., Tognin G., Gavasso S., Bulato C., Iacobelli N.P., Finn J.D., Spiezia L., Radu C., Arruda V.R. (2009). X-Linked Thrombophilia with a Mutant Factor IX (Factor IX Padua). N. Engl. J. Med..

[75] George L.A., Sullivan S.K., Giermasz A., Rasko J.E.J., Samelson-Jones B.J., Ducore J., Cuker A., Sullivan L.M., Majumdar S., Teitel J. (2017). Hemophilia B Gene Therapy with a High-Specific-Activity Factor IX Variant. N. Engl. J. Med..

[76] Crudele J.M., Finn J.D., Siner J.I., Martin N.B., Niemeyer G.P., Zhou S., Mingozzi F., Lothrop C.D., Arruda V.R. (2015). AAV Liver Expression of FIX-Padua Prevents and Eradicates FIX Inhibitor without Increasing Thrombogenicity in Hemophilia B Dogs and Mice. Blood.

[77] Samelson-Jones B.J., Finn J.D., George L.A., Camire R.M., Arruda V.R. (2019). Hyperactivity of Factor IX Padua (R338L) Depends on Factor VIIIa Cofactor Activity. JCI Insight.

[78] Samelson-Jones B.J., Finn J.D., Raffini L.J., Merricks E.P., Camire R.M., Nichols T.C., Arruda V.R. (2021). Evolutionary Insights into Coagulation Factor IX Padua and Other High-Specific-Activity Variants. Blood Adv..

[79] Robinson M.M., George L.A., Carr M.E., Samelson-Jones B.J., Arruda V.R., Murphy J.E., Rybin D., Rupon J., High K.A., Tiefenbacher S. (2021). Factor IX Assay Discrepancies in the Setting of Liver Gene Therapy Using a Hyperfunctional Variant Factor IX-Padua. J. Thromb. Haemost..

[80] U.S. National Library of Medicine A Study to Evaluate the Efficacy and Safety of Factor IX Gene Therapy with PF-06838435 in Adult Males with Moderately Severe to Severe Hemophilia B (BENEGENE-2). ClinicalTrials.gov Identifier: NCT03861273. NCT03861273.

[81] Almeida-Porada G. (2021). A New “FIX” for Hemophilia B Gene Therapy. Blood.

[82] Nair N., De Wolf D., Nguyen P.A., Pham Q.H., Samara-Kuko E., Landau J., Blouse G.E., Chuah M.K., VandenDriessche T. (2021). Gene Therapy for Hemophilia B Using CB 2679d-GT: A Novel Factor IX Variant with Higher Potency than Factor IX Padua. Blood.

[83] Kapugi M., Cunningham K. (2019). Corticosteroids. Orthop. Nurs..

[84] Smith E., Blomberg P. (2017). Gene therapy—From idea to reality. Lakartidningen.

[85] Lippi G., Favaloro E.J. (2020). Gene therapy for hemophilias: The end of phenotypic testing or the start of a new era?. Blood Coagul. Fibrinolysis.

[86] Batty P., Lillicrap D. (2021). Hemophilia Gene Therapy: Approaching the First Licensed Product. Hemasphere.

[87] Iriart J.A.B. (2019). Precision medicine/personalized medicine: A critical analysis of movements in the transformation of biomedicine in the early 21st century. Cad. Saude Publica.

[88] Baynam G., Molster C., Bauskis A., Kowal E., Savarirayan R., Kelaher M., Easteal S., Massey L., Garvey G., Goldblatt J. (2017). Indigenous Genetics and Rare Diseases: Harmony, Diversity and Equity. Adv. Exp. Med. Biol..

[89] Skinner M.W., Nugent D., Wilton P., O’Mahony B., Dolan G., O’Hara J., Berntorp E. (2020). Achieving the unimaginable: Health equity in haemophilia. Haemophilia.

